# Deciphering the genomic landscape of novel *Acinetobacter* non-*baumannii* lineages causing neonatal septicemia: carbapenem resistance and virulence

**DOI:** 10.3389/fmicb.2026.1889965

**Published:** 2026-08-24

**Authors:** Subhasree Roy, Daichi Morita, Tanusree Das, Sanjib Das, Souvik Nandy, Bijan Saha, Sulagna Basu

**Affiliations:** 1 Division of Bacteriology, ICMR National Institute for Research in Bacterial Infections (ICMR-NIRBI), Kolkata, India; 2 Department of Microbiology, Graduate School of Biomedical and Health Sciences, Hiroshima University, Hiroshima, Japan; 3 Department of Neonatology, Institute of Post-Graduate Medical Education & Research and SSKM Hospital, Kolkata, India

**Keywords:** *Acinetobacter* non-*baumannii*, carbapenem resistance, India, NDM-1, novel sequence type, OXA-58-like, virulence, WGS

## Abstract

**Background:**

*Acinetobacter* non-*baumannii* (Anb) species are reported worldwide to cause infections in both adults and neonates, although less frequently than *Acinetobacter baumannii*. However, limited information is available on their genomic diversity, resistance mechanisms, and virulence potential. This study investigates novel Anb isolates causing neonatal septicemia in India to characterize their resistance and pathogenic traits.

**Methods:**

Anb isolates from neonatal blood cultures (2007–2025) were identified by VITEK2 Compact system, MALDI-TOF MS, and Whole-genome sequencing (WGS). Antimicrobial susceptibility was tested by VITEK2. Genomic analysis included MLST, resistome, virulome, plasmid typing, integrons, and core-genome phylogeny analysis. *In vitro* and *in vivo* studies assessed pathogenic potential of Anb species.

**Results:**

Anb infections were low (11%) among the neonates during the study period. WGS revealed 11 novel Sequence Types (STs) which include *A. indicus, A. variabilis, A. schindleri*, and *A. bereziniae*. Six out of these eleven Anbs harbored carbapenemases such as *bla*_NDM–1_ and/or *bla*_OXA–58–like_ genes (*bla*_OXA–58_, *bla*_OXA–420_). *bla*_NDM–1_ was acquired via Tn*125* transposon. IS*Aba125* was located upstream of *bla*_NDM–1_, and a conserved structure extending to IS*91* family transposase was detected in *bla*_NDM–1–_harboring genomes. *bla*_OXA–58–like_ genes were found to be associated with IS*Aba3*. Most carbapenemases were likely located on chromosome. Class 1 integrons carrying multiple antimicrobial resistance genes (ARGs) and diverse plasmid replicase families were detected in Anbs. Core genome phylogeny showed that the study Anbs were not closely related to the global Anbs. *In vitro* virulence-associated assays (biofilm formation, surface motility, adherence/invasion, apoptosis) and *in vivo* lethality in murine infection model showed reduced pathogenicity, reinforcing earlier observations that Anb species are generally less virulent than *A. baumannii*. Several virulence factors (VFs) were detected; however, no clear correlation was observed between virulence genes, *in vitro* pathogenicity, and *in vivo* lethality.

**Conclusion:**

These results indicate the multifactorial nature of Anb pathogenicity and the current limitations of knowledge of its VFs. However, the presence of numerous VFs suggests a capacity to cause disease, particularly in vulnerable host populations such as neonates. Furthermore, the presence of multiple ARGs indicates a strong potential for persistence and dissemination in hospital environments with high antibiotic pressure. Overall, these findings underscore the importance of continued AMR surveillance, genome characterization and further investigations into Anb pathogenicity.

## 1 Introduction

The genus *Acinetobacter* comprises a highly diverse group of bacteria, recognized as a major opportunistic nosocomial pathogen. These species are ubiquitous in the environment, found in water, soil, sewage, vegetables, animals and humans (Al Atrouni et al., 2016). Currently, more than 70 *Acinetobacter* species have been identified,^1^ with *A. baumannii* being the most clinically significant (Antunes et al., 2014; Whiteway et al., 2022). However, infections have also been reported to be caused by other less common *Acinetobacter* non-*baumannii* (Anb) species such as *A. nosocomialis*, *A. pittii*, *A. bereziniae*, *A. johnsonii*, *A. junii*, *A. lwoffii*, *A. soli*, *A. ursingii*, *A. indicus*, and *A. schindleri* (Kuo et al., 2010; Visca et al., 2011; Kee et al., 2018; Sheck et al., 2023). These pathogens are also increasingly implicated in neonatal sepsis worldwide including India (Chatterjee et al., 2016; Zarrilli et al., 2018; Nazir, 2019; Pillay et al., 2024; Roy et al., 2024).

Despite species variation, a shared feature of all *Acinetobacter* species is the acquisition of multiple transferable resistance genes across diverse antibiotic classes including extended-spectrum β-lactamases (ESBLs), chromosomal OXA-type β-lactamases, aminoglycoside-modifying enzymes and carbapenemases (Périchon et al., 2014; Baraka et al., 2020). Carbapenems are typically the drugs of choice for severe *Acinetobacter* infections, however, the global emergence of carbapenem-resistant *Acinetobacter* isolates is alarming (Sheck et al., 2023). In the World Health Organization’s, 2024 Priority Pathogens List, carbapenem-resistant *A. baumannii* is placed in the Critical group, signaling an urgent need for novel antimicrobial development (Tacconelli, n.d.; World Health Organization, 2024) . The threat is even more severe in neonates where therapeutic options are extremely limited.

While carbapenem resistance in *A. baumannii* has been extensively documented, investigations of resistance mechanisms in Anb species have been relatively limited (Sheck et al., 2023). Prior studies indicated that carbapenem resistance in Anb was primarily mediated by class B metallo-β-lactamases (MBLs), notably NDM and IMP (Yamamoto et al., 2013; Montaña et al., 2018; Tang et al., 2021; Mo et al., 2023) and class D carbapenemases including OXA-23, OXA-24/40, and OXA-58 (Mendes et al., 2009; Cayô et al., 2016; Strateva et al., 2018; Fávaro et al., 2019). Among these, OXA-58 has been widely reported in Anb isolates worldwide, either alone (Mendes et al., 2009; Cayô et al., 2016; Strateva et al., 2018; Fávaro et al., 2019) or in combination with MBLs such as NDM-1 and IMP variants (Ang et al., 2016; Feng et al., 2016; Chen et al., 2019; Jiang et al., 2019; Iimura et al., 2020; Kim et al., 2024). A recent study by Migliaccio et al. (2023) analyzed 837 genomes of 72 *Acinetobacter* species, and identified a wide range of antimicrobial resistance genes conferring resistance to multiple antimicrobial classes. Furthermore, MBL genes (*bla*_GIM–1_, *bla*_IMP–1,–4,–14,–19,–34,_ *bla*_NDM–1,–16_, *bla*_VIM–2,–4_) and class D carbapenemase genes (*bla*_OXA–23_, *bla*_OXA–40_, *bla*_OXA–58_ family) were also detected across various *Acinetobacter* genus infrequently, highlighting that Anb isolates contain diverse reservoirs of antimicrobial resistance genes and may contribute to infections.

Despite the increasing clinical relevance of Anb species, there is still limited understanding of their antimicrobial resistance mechanisms and virulence profile compared to *A. baumannii*. As these emerging pathogens begin to mirror *A. baumannii* in both resistance and clinical impact, there is an urgent need for detailed molecular characterization of these pathogens. This study investigates clinical Anb isolates obtained from cases of neonatal septicemia, with the aim of identifying sequence types (STs), mechanisms of carbapenem resistance, associated mobile genetic elements and the pathogenic potential of these Anb species. Additionally, core genome phylogenetic analysis was also performed to assess the genomic variability of these Anb isolates (included in the study) with other Anb strains reported globally.

## 2 Materials and methods

### 2.1 Identification and antibiotic susceptibility of *Acinetobacter* isolates

*Acinetobacter* strains were isolated from blood of septicemic neonates (2007–2025), admitted to the Neonatal Intensive Care Unit (NICU) of a tertiary care hospital in Kolkata, India. Strains were isolated from blood samples as a part of routine diagnosis of sepsis in neonates and archived as 20% glycerol stocks in −80°C. One milliliter of blood was drawn from the peripheral vein of septicemic neonates under strict aseptic conditions, followed by inoculation in BD Bactec Peds Plus Vials (Becton Dickinson, Sparks, MD). Blood culture was carried out on an automated Bactec 9050 system (Becton Dickinson, Franklin Lakes, NJ). Any culture that flagged positive was subcultured on appropriate media based on Gram’s stain: MacConkey agar (Difco Laboratories, Detroit, MI, United States) and 5% sheep blood agar (Difco Laboratories) for Gram-negative isolates. Clinical data was collected from the hospital registers.

Identification was carried out by VITEK2 Compact system and MALDI-TOF MS (BioMérieux, France) followed by OXA-51 PCR (Turton et al., 2006). Antimicrobial susceptibility testing was done using VITEK2 Compact system.

### 2.2 Genomic characterization of *Acinetobacter* non-*baumannii* strains using whole-genome sequencing

Total genomic DNA was extracted using the Wizard DNA Purification Kit (Promega), and quantified using the Qubit 3.0 (Thermo Fisher Scientific). DNA libraries were prepared for paired end sequencing (2 × 250 cycles) using Nextera XT (Illumina Inc., San Diego, CA). WGS was performed using the Illumina NovaSeq 6000 platform (Illumina Inc., San Diego, CA). Quality control of raw reads included Fastqc (v0.11.8), and quality and adaptor trimming were performed using Trimgalore (v0.6.4). Reads were assembled in contigs using Shovill (v0.9.0) (.fasta). Assembly metrics were evaluated using Quast (v5.0.2) (Gurevich et al., 2013). Genomes (contigs) were annotated using Prokka (v1.14.5) (Seemann, 2014) and the resulting .gbk file was viewed in Artemis (genome viewer, Sanger, United Kingdom) (Carver et al., 2012). Using the WGS short read data the following analysis were carried out-

Assembled genomes were submitted to the pubMLST database to get new ST numbers for the novel Anbs (Jolley et al., 2018). Antimicrobial resistance genes (ARGs), virulence factors, and integrons, were identified using different online tools, including ABRicate, ResFinder (Bortolaia et al., 2020), CARD (Alcock et al., 2023). VFDB (Chen et al., 2016), and INTEGRALL (Moura et al., 2009) respectively. Replicases were identified using BLAST against the *Acinetobacter* Plasmid Typing database (> 95% identity) (Lam et al., 2023). The location of ARGs either on plasmid or chromosome was predicted using MOB-Suite (v3.0.3) analysis (Robertson and Nash, 2018). Translated nucleotide sequences of *gyrA* and *parC* genes was aligned to detect the amino acids at position 81 of GyrA and 84 of ParC, within the QRDR of *Acinetobacter* spp.

### 2.3 Core genome phylogenomic analysis

The core genome analysis was performed to compare the studied Anb isolates with publicly available Anb genomes originating from diverse hosts and environmental sources across different countries and years. The analysis was performed using Panaroo v1.3.3 (Tonkin-Hill et al., 2020) with pangenome sub workflow of Bactopia v3.0.0 (Petit and Read, 2020), using default parameters except for –use_panaroo and –panaroo_alignment pan. The pairwise SNP distance matrix was calculated with snp-dists 0.8.2 [Seemann T snp-dists - Pairwise SNP distance matrix from a FASTA sequence alignment, (GitHub)] with the default parameters. A phylogenetic tree was generated using IQ-TREE v2.2.2.7 (Nguyen et al., 2015) and visualized using the Interactive Tree of Life v.6.5 (iTOL) (Letunic and Bork, 2024).

### 2.4 Transmissibility of carbapenemase genes

The transmissibility of *bla*_NDM–1_ and *bla*_OXA–58–like_ genes was assessed through solid mating and liquid mating conjugation assays using *E. coli* J53 Az*^r^* (resistant to sodium azide at 100 mg/L). The donor and recipient cultures were mixed in 1:2, 1:5, and 1:10 ratio and were plated on Luria Bertani (LB) agar plates supplemented with 0.25 mg/L meropenem and 100 mg/L sodium-azide (Sigma-Aldrich, St. Louis, MO, United States) to screen transconjugants (TCs) (Chatterjee et al., 2016).

### 2.5 *In vitro* virulence assays

Several *in vitro* virulence assays were performed with the Anb strains to evaluate bacterial survival, persistence, cellular adherence, intracellular entry, and percentage of live/cell death during infection. Biofilm formation was assessed using overnight bacterial suspension in 96-well plates with absorbance measured at 595 nm. Strong biofilm formation was considered when the readings were at least three times greater than the negative control. *Vibrio cholerae* HyR0114 and *A. baumannii* ATCC 19606 were used as high biofilm producer while *V. cholerae* HyRS01 was used as low biofilm producer (Chowdhury et al., 2016; Mukherjee et al., 2020). Surface motility was assessed on LB agar (0.3%) and the radii were measured at 24–72 h. *V. cholerae* 01 0395 was used as positive control (Chowdhury et al., 2016; Carretero-Ledesma et al., 2018). Growth under iron-limiting conditions was measured in Mueller-Hinton broth with or without the iron chelator 2,2’-bipyridyl (150 μM) and OD_595_nm was determined at 1, 2, 3, 4, and 24 h (Carretero-Ledesma et al., 2018). For adherence, A549 epithelial cells were infected with Anbs at MOI 100 for 90 min. Invasion was determined following gentamicin treatment (300 μg/mL) for 2 h followed by CFU enumeration (Lázaro-Díez et al., 2016). *Shigella flexneri* 2a2457T and *A. baumannii* ATCC 19606 were used as positive and negative controls, respectively. Apoptosis was quantified by staining with annexin V-FITC/PI and analysis was done using flow cytometry. Detail methods of *in vitro* virulence assays are provided in Supplementary file.

### 2.6 *In vivo* mortality assay

BalB/C female mice weighing 18–23 g were used in this study. For i.p. inoculation in mice, freshly cultured inoculum was prepared for each experiment from frozen stocks of Anbs. Mice (*n* = 3) were inoculated with 1 x 10^8^ CFU/mL of Anbs (all 11 studied Anb strains were examined) in 100μl saline and monitored for survival daily for 7 days. Experiments were performed in duplicate on separate days (Ku et al., 2019). Survival data were analyzed using Kaplan-Meier curves in GraphPad Prism v8.0.1.

## 3 Results and discussion

### 3.1 Identification, antibiotic susceptibility and MLST of *Acinetobacter* non-*baumannii*

A total of 213 *Acinetobacter* spp., were isolated from blood of septicemic neonates. Of these, 190 were identified as *A. baumannii* by MALDI-TOF MS and *bla*_OXA–51_ gene presence. The rest (*n* = 23) did not have *bla*_OXA–51_ in them. Of them, 10 isolates were initially identified as Anbs by VITEK but could not be revived later. The remaining 13 isolates were confirmed as *Acinetobacter* non-*baumannii* (Anb) by WGS. Overall, 23 out of 213 (11%) *Acinetobacter* spp., were detected as Anbs during the study period (2007–2025). Further analyses were limited to the WGS confirmed Anb isolates only.

MLST analysis revealed 12 novel sequence types (STs) among the 13 Anb isolates, including two *A. schindleri* (ST-1485, ST-2161), one *A. bereziniae* (ST-1503), four *A. indicus* (ST-2168, ST-2169, ST-2172, ST-2174), four *A. variabilis* (ST-2170, ST-2171, ST-2173, ST-2175) and two *A. pittii* (ST-1451). As the two *A. pittii* isolates have been reported previously (Roy et al., 2024), this study focused on the remaining 11 novel Anb isolates (Table 1). GoeBURST analysis showed that all 11 novel STs were singletons, indicating no allelic relatedness to the existing STs.

**TABLE 1:** Characterization of novel *Acinetobacter* non-*baumannii* strains (*n* = 11) isolated from blood of septicemic neonates.

Variables	Strain information										
Strain number	A_114	A_127	A_135	A_137	A_139	A_148	A_154	A_164	A_165	A_186	A_187
Organisms	*A. bereziniae*	*A. indicus*	*A. schindleri*	*A. indicus*	*A. variabilis*	*A. variabilis*	*A. schindleri*	*A. indicus*	*A. variabilis*	*A. indicus*	*A. variabilis*
Year of isolation	2009	2011	2011	2011	2011	2012	2013	2013	2013	2016	2016
Sequence type	ST1503	ST2168	ST2161	ST2169	ST2170	ST2171	ST1485	ST2172	ST2173	ST2174	ST2175
Size (bp)	4,716,380	3,241,328	3,555,134	3,147,993	3,447,940	3,442,244	3,674,376	3,052,069	3,351,466	3,318,073	3,741,253
G+C content (%)	38.1	45.0	42.0	45.5	42.2	42.2	41.9	45.8	41.9	45.3	41.8
Antimicrobial susceptibility profile (MIC in mg/L)	CAZ (> = 64, R); FEP (> = 64, R); ATZ (> = 64, R); DOR (0.5, S); IMP (0.5, S); MEP ( ≤ 0.25, S); AMK (> = 64, R); GEN (> = 16, R); CIP (> = 4, R); MINO ( < = 1, S); TGC (0.125, S); COL (3, R	CAZ (> = 64, R); FEP (32, R); ATZ (> = 64, R); DOR ( ≤ 0.12, S); IMP (0.5, S); MEP ( ≤ 0.25, S); AMK (16, S); GEN (> = 16, R); CIP (> = 4, R); MINO ( < = 1, S); TGC ( ≤ 0.5, S); COL ( ≤ 0.5, S)	CAZ (> = 64, R); FEP (16, R); ATZ (16, R); DOR (> = 8, R); IMP (> = 16, R); MEP (> = 16, R); AMK (16, S); GEN (> = 16, R); CIP (> = 4, R); MINO ( = 1, S); TGC ( ≤ 0.5, S); COL ( ≤ 0.5, S)	CAZ (> = 64, R); FEP (2, S); ATZ (4, S); DOR (> = 16, R); IMP (> = 16, R); MEP (> = 16, R); AMK (2, S); GEN (> = 16, R); CIP (2, R); MINO ( = 1, S); TGC ( ≤ 0.5, S); COL ( ≤ 0.5, S)	CAZ (> = 64, R); FEP (> = 64, R); ATZ (> = 64, R); DOR (0.5, S); IMP ( ≤ 0.25, S); MEP (1, S); AMK ( = 2, S); GEN ( = 1, S); CIP (> = 4, R); MINO ( = 1, S); TGC ( = 0.5, S); COL ( = 0.5, S)	CAZ (> = 64, R); FEP (32, R); ATZ (> = 64, R); DOR ( ≤ 0.12, S); IMP ( ≤ 0.25, S); MEP ( ≤ 0.25, S); AMK (16, S); GEN ( = 1, S); CIP (4, R); MINO ( = 1, S); TGC ( ≤ 0.5, S); COL ( ≤ 0.5, S)	CAZ (4, S); FEP ( = 1, S); ATZ (32, R); DOR ( ≤ 0.12, S); IMP ( ≤ 0.25, S); MEP ( ≤ 0.25, S); AMK (16, S); GEN (2, S); CIP (> = 4, R); MINO ( = 1, S); TGC ( ≤ 0.5, S); COL ( ≤ 0.5, S)	CAZ (> = 64, R); FEP (32, R); ATZ (2, S); DOR (> = 8, R); IMP (8, R); MEP (> = 16, R); AMK (16, S); GEN ( = 1, S); CIP (1, S); MINO ( = 1, S); TGC ( ≤ 0.5, S); COL ( ≤ 0.5, S)	CAZ (> = 64, R); FEP (> = 64, R); ATZ (> = 64, R); DOR (> = 8, R); IMP (> = 16, R); MEP (> = 16, R); AMK (> = 64,R); GEN (> = 16, R); CIP (> = 4, R); MINO ( = 1, S);TGC ( = 0.5, S); COL ( = 0.5, S)	CAZ (> = 64, R); FEP (> = 64, R); ATZ (16, R); DOR (> = 8, R); IMP (4, R); MEP (4, R); AMK (16, S); GEN (8, R); CIP (> = 4, R); MINO ( = 1, S); TGC ( ≤ 0.5, S); COL ( ≤ 0.5, S)	CAZ (4, S); FEP (> = 64, R); ATZ (16, R); DOR ( ≤ 0.12, S); IMP ( ≤ 0.25, S); MEP ( ≤ 0.25, S); AMK ( = 2, S); GEN ( = 1, S); CIP ( ≤ 0.25, S); MINO ( = 1, S); TGC ( ≤ 0.5, S); COL ( ≤ 0.5, S)
β-lactamase (bla)	*bla*_OXA–355_ (class D OXA-228-like)	*bla*_PER–7_	*bla*_OXA–276_ (class D OXA-134-like)	nil	*bla*_VEB–1_	*bla*_PER–7_	*bla*_OXA–277_ (class D OXA-134-like)	nil	nil	*bla*_CARB–2_	*bla*_TEM–1B_, *bla*_VEB–1_
Aminoglycoside and fluoroquinolone resistance genes	*aph(3″)-Ib, aac(3)-IId, aph(3′)-VI* *aph(6)-Id*	*aac(3)-IId, aph(6)-Id, aph(3″)-Ib, aph (3′)-VI*	*ant(2″)-Ia, aph(3′)-VI, aph(3″)-Ib, aph(6)-Id*	*ant(2″)-Ia, aac(6′)-Ib3, aph(3′)-VI, aph(3″)-Ib, aph(6)-Id, aac(6′)-Ib-cr*	*aph(3″)-Ib, aph(6)-Id*	*aac(6′)-Ib3, aph(3′)-Via, aac(6′)-Ib-cr*	*aac(3)-IId, aac(6′)-Ib3, aph(3′)-Via, aac(6′)-Ib-cr*	*aph(3′)-VI*	*armA, aph(3′)-VI*	*ant(2″)-Ia, aph(3″)-Ib, aph(6)-Id* *aph(3′)-VI*	*aph(6)-Id, aph(3′)-VIa, aph(3″)-Ib, aac(3)-IIa*
carbapenemases	nil	*bla*_OXA–420_	*bla*_NDM–1_	*bla*_NDM–1_	nil	nil	nil	*bla*_NDM–1_, *bla*_OXA–58_	*bla*_NDM–1_	*bla*_NDM–1_, *bla*_OXA–420_	nil
Other resistance genes	*mph*(E) & *msr*(E) (macrolide); *sul1, sul2* (sulphonamide); *floR* (chloramphenicol, florfenicol); *qacE* (quaternary ammonium compounds); *arr-3* (rifampicin), *dfrA1* (trimethoprim)	*sul1* & *sul2* (sulphonamide)	*mph*(E), *msr*(E) (macrolide); *sul2* (sulphonamide); *floR, cmlB1* (chloramphenicol, florfenicol), *tet(39)* (tetracycline, doxycycline)	*mph*(E), *msr*(E) (macrolide); *sul2* (sulphonamide); *floR* (chloramphenicol, florfenicol), *arr-3* (rifampicin), *dfrA19* (trimethoprim)	*mph*(E), *msr*(E) (macrolide); *sul1*& *sul2* (sulphonamide); *floR* (chloramphenicol, florfenicol), *qacE* (quaternary ammonium compounds), *tet(A*) (tetracycline, doxycycline)	*sul1* (sulphonamide); *qacE* (quaternary ammonium compounds), *tet(39)* (tetracycline, doxycycline), *arr-3* (rifampicin), *dfrA19* (trimethoprim)	*mph*(E), *msr*(E) (macrolide); *sul1* (sulphonamide); *qacE* (quaternary ammonium compounds), *arr-3* (rifampicin)	*mph*(E) & *msr*(E) (macrolide); *sul2* (sulphonamide)	*mph*(E) & *msr*(E) (macrolide)	*mph*(E) & *msr*(E) (macrolide); *sul2* (sulphonamide), *floR* (chloramphenicol, florfenicol), *tet(G)* (tetracycline, doxycycline)	*mph*(E) & *msr*(E) (macrolide); sul1 (sulphonamide), *dfrA1* (trimethoprim), *qacE* (quaternary ammonium compounds), *tet(A)* (tetracycline, doxycycline)
GyrA/ParC mutations	*gyrA*(S81L), *parC*(S84Y)	*gyrA*(S81F), *parC*(S84L)	*gyrA*(S81F), *parC*(S84L)	*gyrA*(S81Y), *parC*(S84L)	*gyrA*(S81F), *parC*(S84L)	*gyrA*(S81F), *parC*(S84L)	*gyrA*(S81F), *parC*(S84L)	*gyrA*(S81F)	*gyrA*(S81F), *parC*(S84L)	*gyrA*(S81F), *parC*(S84L)	*gyrA*(S81F)
*Acinetobacter* Plasmid Typing database replicases	Rep_3 family protein R3-T12/ Rep_3 family protein R3-T14/ Rep_3 family protein R3-T15/ Rep_3 family protein R3-T29/ Rep_3 family protein R3-T34/ Rep_3 family protein R3-T123/ Rep_3 family protein R3-T139/ RepPriCT_1 family protein RP-T9	Rep_3 family protein R3-T17/ Rep_3 family protein R3-T20/ Rep_3 family protein R3-T25/ Rep_3 family protein R3-T26/ Rep_3 family protein R3-T26*/ Rep_3 family protein R3-T45/ Rep_3 family protein R3-T90	Rep_3 family protein R3-T14/ Rep_3 family protein R3-T26/ Rep_3 family protein R3-T26*/ Rep_3 family protein R3-T33/ Rep_3 family protein R3-T80	Rep_3 family protein R3-T20/ Rep_3 family protein R3-T26/ Rep_3 family protein R3-T26*/ Rep_3 family protein R3-T62/ RepPriCT_1 family protein RP-T4	Rep_3 family protein R3-T25/ Rep_3 family protein R3-T29/ Rep_3 family protein R3-T45/ Rep_3 family protein R3-T90/ Rep_3 family protein R3-T134	Rep_3 family protein R3-T14/ Rep_3 family protein R3-T15/ Rep_3 family protein R3-T17/ Rep_3 family protein R3-T25/ Rep_3 family protein R3-T62/ Rep_3 family protein R3-T90	Rep_3 family protein R3-T1/ Rep_3 family protein R3-T14/ Rep_3 family protein R3-T26/ Rep_3 family protein R3-T26*/ Rep_3 family protein R3-T62/ Rep_3 family protein R3-T90	Rep_3 family protein R3-T10/ Rep_3 family protein R3-T11/ Rep_3 family protein R3-T15/ Rep_3 family protein R3-T65	Rep_3 family protein R3-T29/ Rep_3 family protein R3-T60/ Rep_3 family protein R3-T65/ Rep_3 family protein R3-T80	Rep_3 family protein R3-T25/ Rep_3 family protein R3-T26/ Rep_3 family protein R3-T26*/ Rep_3 family protein R3-T45/ Rep_3 family protein R3-T90/ Rep_3 family protein R3-T145	Rep_3 family protein R3-T14/ Rep_3 family protein R3-T25/ Rep_3 family protein R3-T45/ Rep_3 family protein R3-T76/ Rep_3 family protein R3-T80/ Rep_3 family protein R3-T90/ Rep_3 family protein R3-T100

CAZ, ceftazidime; FEP, cefepime; ATZ, aztreonam; DOR, doripenem; IMP, imipenem; MEM, meropenem; AMK, amikacin; GEN, gentamicin; PIP, piperacillin; CIP, ciprofloxacin; MINO, minocycline; TGC, tigecycline; COL, colistinS, Serine; L, Leucine; Y, Tyrosine; F, Phenylalanine.

Antibiotic susceptibility test showed high resistance to ceftazidime, cefepime, ciprofloxacin, aztreonam (9/11 for each), and gentamicin (6/11). Five isolates were detected as carbapenem-resistant (imipenem or meropenem or doripenem), while amikacin, minocycline, tigecycline and colistin resistance were very low (Table 1).

### 3.2 Demographic and clinical features of the patients

Among the 11 neonates with septicemic caused by Anb strains (including both Early-onset and Late-onset cases), clinical details were available for 8 neonates (Table 2). Most of the neonates were male, pre-term (gestational age < 37 weeks), had low birth weight (<2,500 g), and were delivered by lower uterine caesarean section. Empirical broad-spectrum antibiotics (a combination of piperacillin-tazobactam and amikacin) were given to all neonates after blood samples were collected for culture. Additional antibiotics including, ofloxacin, netilmicin, gentamicin or a combination of amoxicillin and clavulanic acid were administered in selected cases. Three neonates died while the others recovered and were discharged.

**TABLE 2:** Demographic, clinical, and treatment data of neonates with *Acinetobacter* non-*baumannii* sepsis.

Strain no	Organism	Date of birth	Sex	Gestational age (weeks)	Birth weight (g)	Intramural/ extramural birth	Baby in NICU	Mode of delivery	Ventilation	Onset of Sepsis	Administration of Antibiotics	Outcome
A_127	*A. indicus*	Dec, 2010	Female	31	1,229	Intramural	Yes	LUCS	Yes	EOS	Inj piptazo + amikacin	Discharge
A_135	*A. schindleri*	Feb, 2011	Male	38	2,600	Extramural	Yes	LUCS	No	LOS	Inj piptazo + amikacin	Death
A_137	*A. indicus*	April, 2011	Male	37	2,550	Intramural	Yes	NVD	No	LOS	Inj piptazo + amikacin, ofloxacin + netilmicin	Discharge
A_139	*A. variabilis*	Aug, 2011	Male	31	1,250	Intramural	Yes	NVD	Yes	EOS	Inj piptazo + amikacin, co-amoxiclav + gentamicin	Discharge
A_148	*A. variabilis*	Jan, 2012	Male	31	1,404	Intramural	Yes	LUCS	No	EOS	Inj piptazo + amikacin	Discharge
A_154	*A. schindleri*	Feb, 2013	Male	38	2,750	Extramural	Yes	NVD	Yes	EOS	Inj piptazo + amikacin	Death
A_164	*A. indicus*	Nov, 2013	Male	28	953	Extramural	Yes	LUCS	Yes	EOS	Inj piptazo + amikacin	Death
A_165	*A. variabilis*	Nov, 2013	Female	31	1,320	Extramural	Yes	LUCS	No	LOS	Inj piptazo + amikacin, co-amoxiclav	Discharge

Among the 11 neonates with septicemia caused by *Acinetobacter* non-*baumannii* strains, clinical details were available for 8 neonates. Pre-term (gestational age < 37 weeks); low birth weight (<2,500 g); intramural birth (born at the hospital); extramural birth (born outside the hospital); LUCS- lower uterine cesarean section; NVD- normal vaginal delivery; EOS-Early-onset sepsis (sepsis is manifested within 72 h of life); LOS- Late-onset sepsis (sepsis presenting after 72 h of life); Inj**-** injection; piptazo- piperacillin/tazobactam; co-amoxiclav- combination of amoxicillin and clavulanic acid.

### 3.3 Whole genomes sequencing of the novel *Acinetobacter* non-*baumannii* isolates

Several aspects of the genomes were studied and analyzed using WGS.

#### 3.3.1 Resistance genes analysis and replicon typing

The studied Anbs (*n* = 11) harbored several antimicrobial resistance genes (ARGs) of different classes including cephalosporin, carbapenem, aminoglycoside, chloramphenicol, sulfonamide, macrolide, quinolone, trimethoprim, and tetracycline (Table 1). Genes encoding Class A Extended Spectrum-β-lactamases (*bla*_PER–7_, *bla*_VEB–1_) and Class A β-lactamase (*bla*_TEM–1B_) were detected in *A. indicus*, and *A. variabilis*.

Carbapenem resistance was predominantly driven by Class B metallo-β-lactamase (MBLs) *bla*_NDM–1_, detected in five isolates [*A. indicus* (*n* = 3), *A. schindleri* (*n* = 1), *A. variabilis* (*n* = 1)]. While *bla*_NDM–1_ is frequently associated with *A. baumannii* (Rodrigues et al., 2024), reports in Anb species remain limited (Montaña et al., 2018; Tang et al., 2021; Mo et al., 2023). Class D carbapenemases (*bla*_OXA–58–like_ including *bla*_OXA–58_ and *bla*_OXA–420_) were also detected in three *A. indicus* isolates either alone or in combination with *bla*_NDM–1_ as reported earlier (Mendes et al., 2009; Ang et al., 2016; Cayô et al., 2016, 2016; Feng et al., 2016; Strateva et al., 2018; Chen et al., 2019; Fávaro et al., 2019; Jiang et al., 2019; Iimura et al., 2020; Migliaccio et al., 2023; Kim et al., 2024). *bla*_OXA–58_ gene was initially identified in France in 2003 in a multidrug-resistant (MDR) *A. baumannii* (Poirel et al., 2005), however, its presence in Anbs is still rare compared to *A. baumannii*. Intrinsic oxacillinases such as *bla*_OXA–134–like_ (*bla*_OXA–276,_ *bla*_OXA–277_) in *A. schindleri* and *bla*_OXA–228–like_ (*bla*_OXA–355_) in *A. bereziniae* were also identified as previous published report (Migliaccio et al., 2023).

Beyond β-lactamases, genes (*aph(3″)-Ib*, *aph(3′)-VI*, *aph(6)-Id*, *ant(2″)-Ia*, *aac(6′)-Ib3*, *aac(3)-IIa*, and *aac(3)-IId*) encoding aminoglycoside-modifying enzymes were also detected in the studied Anb genomes. A single *A. variabilis* isolate harbored the 16S rRNA methyltransferase gene *armA*, suggesting that high-level aminoglycoside resistance via methylation remains uncommon among Anbs. The earlier study also highlighted the widespread prevalence of aminoglycoside-modifying enzymes as the principal mechanism of aminoglycoside resistance among Anb species, with *aph(6)-Id*, *aph(3″)-Ib* representing the most commonly identified resistance determinants (Baraka et al., 2020). The plasmid-mediated fluoroquinolone resistance gene *aac(6′)-Ib-cr*, rarely reported in Anb (Baraka et al., 2020; Tang et al., 2021), was detected in *A. variabilis*, *A. indicus* and *A. schindleri*.

The studied Anbs also harbored additional ARGs (*sul1*, *sul2*, *tet*, *floR*, *arr-3*, *mph(E)/msr(E)*, *dfrA* variants, *qacE*), as reported in case of *Acinetobacter* spp. (Chatterjee et al., 2016; Yaikhan et al., 2024; Lima et al., 2025). The co-occurrence of disinfectant and antibiotic resistance genes suggests environmental selection pressures and the involvement of mobile genetic elements in resistance gene clustering.

Replicon typing analysis showed the presence of Rep_3 family replicases, including R3-T1, R3-T4, R3-T10, R3-T11, R3-T12, R3-T14, R3-T15, R3-T17, R3-T20, R3-T25, R3-T26, R3-T29, R3-T33, R3-T34, R3-T45, R3-T60, R3-T62, R3-T65, R3-T76 R3-T80, R3-T90, R3-T100, R3-T123, R3-T134, R3-T139, and R3-T145 along with RepPriCT_1 family replicases such as RP-T4 and RP-T9 (Table 1). However, the carbapenemase-harboring contigs lacked identifiable plasmid replicases when BLAST searches against the *Acinetobacter* Plasmid Typing database was done.

MOB-Suite analysis predicted that *bla*_NDM–1_ and *bla*_OXA–58–like_ are likely located on the chromosome in most of the studied genomes of Anb species, consistent with previous reports of chromosome-borne *bla*_NDM–1_ and *bla*_OXA–58–*like*_ in both *A. baumannii* and Anb isolates (Ramirez et al., 2020; Liu et al., 2025). However, in isolate A_135, *bla*_NDM–1_ was identified on a plasmid, lacking plasmid replicases. Furthermore, conjugation experiments were unsuccessful for all studied isolates. Taken together, the *in-silico* prediction of chromosomal localization, the absence of plasmid replicases on carbapenemase-harboring contigs, and the failure of conjugative transfer indicated that *bla*_NDM–1_ and *bla*_OXA–58–like_ were likely chromosomally encoded among the study isolates. Nevertheless, long-read sequencing would be required to confirm the genomic location of these genes.

### 3.3.2 Genetic structure of *bla*_NDM–1_

**FIGURE 1:**
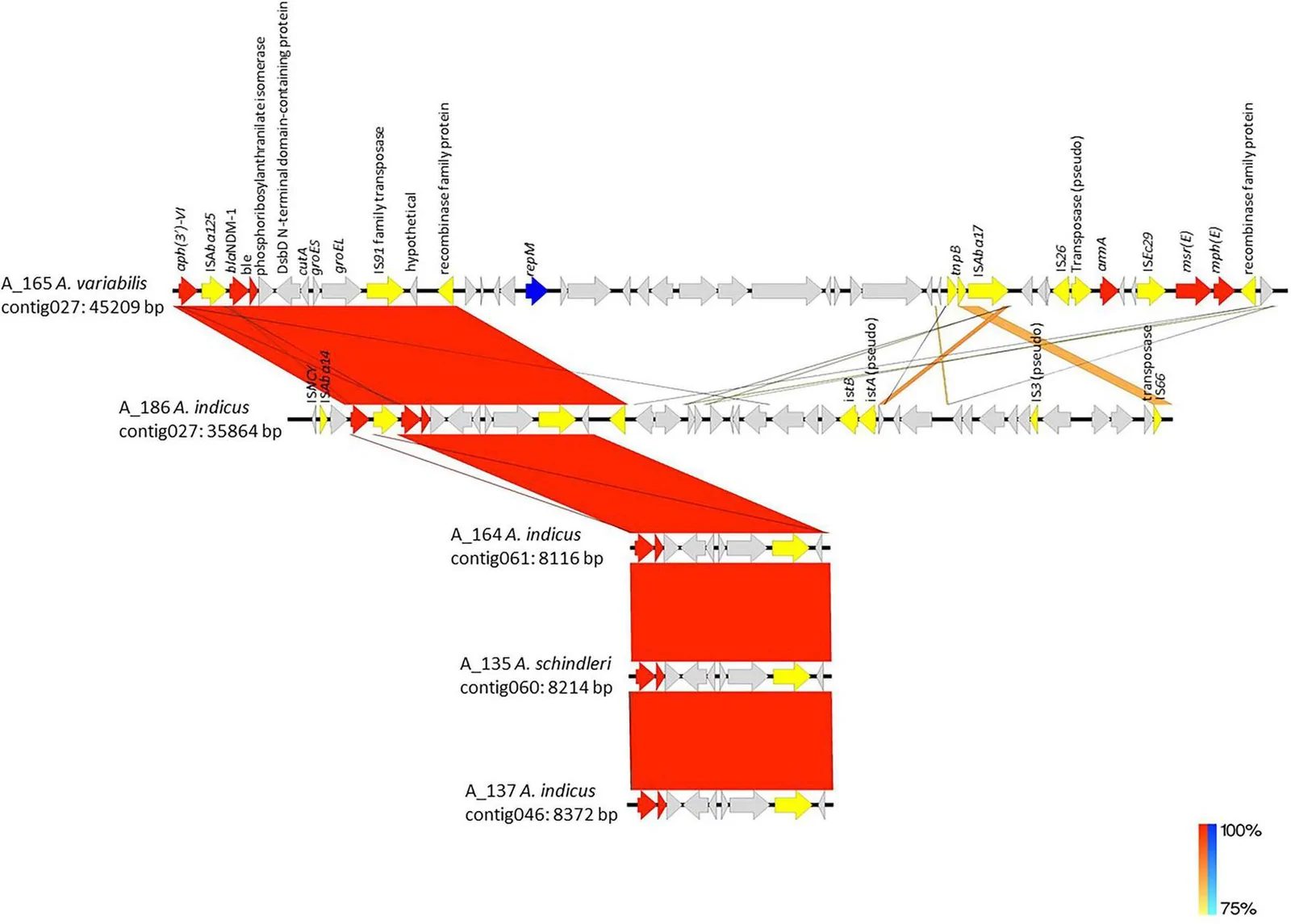
The schematic diagram of the regions surrounding *bla*_NDM–1_ among *Acinetobacter* non-*baumannnii* strains. Genes and their corresponding transcription orientations are indicated by horizontal arrows. *bla*_NDM–1_ is propagated by the Tn*125* transposon, in which these sequences are flanked by IS*Aba125*.

The *bla*_NDM–1_-possessing Anbs (*n* = 5) exhibited identical structures surrounding the gene consistent with structures widely identified in *Acinetobacter* spp., that have acquired *bla*_NDM–1_ via the Tn*125* transposon (Poirel et al., 2012; Ramirez et al., 2020; Liu et al., 2025). The upstream region of *bla*_NDM–1_ contains IS*Aba125*, while the downstream region includes *ble*_*MBL*_ (encoding bleomycin resistance), followed by *trpF* (phosphoribosylantranilate isomerase), *dsbD* (protein disulfide reductase), *cutA1* (periplasmic divalent cation tolerance protein), *groES/groEL* (encoding chaperonin), and transposases belonging to IS*91* (Figure 1). It was found that among the *bla*_NDM–1_ positive isolates available in the database (originating from diverse hosts and environmental sources across different countries and years), most possessed a conserved genetic structure extending from *bla*_NDM–1_ to IS*91* family transposase. This indicates that the genomic regions surroundings the Tn*125*-related structure are highly conserved and maintained in Anb population. *bla*_OXA–58–*like*_ genes were found to be associated with IS*Aba3* elements, which are known to promote the mobilization and expression of OXA-58-like carbapenemases (Ramirez et al., 2020).

#### 3.3.3 Integron analysis

**FIGURE 2:**
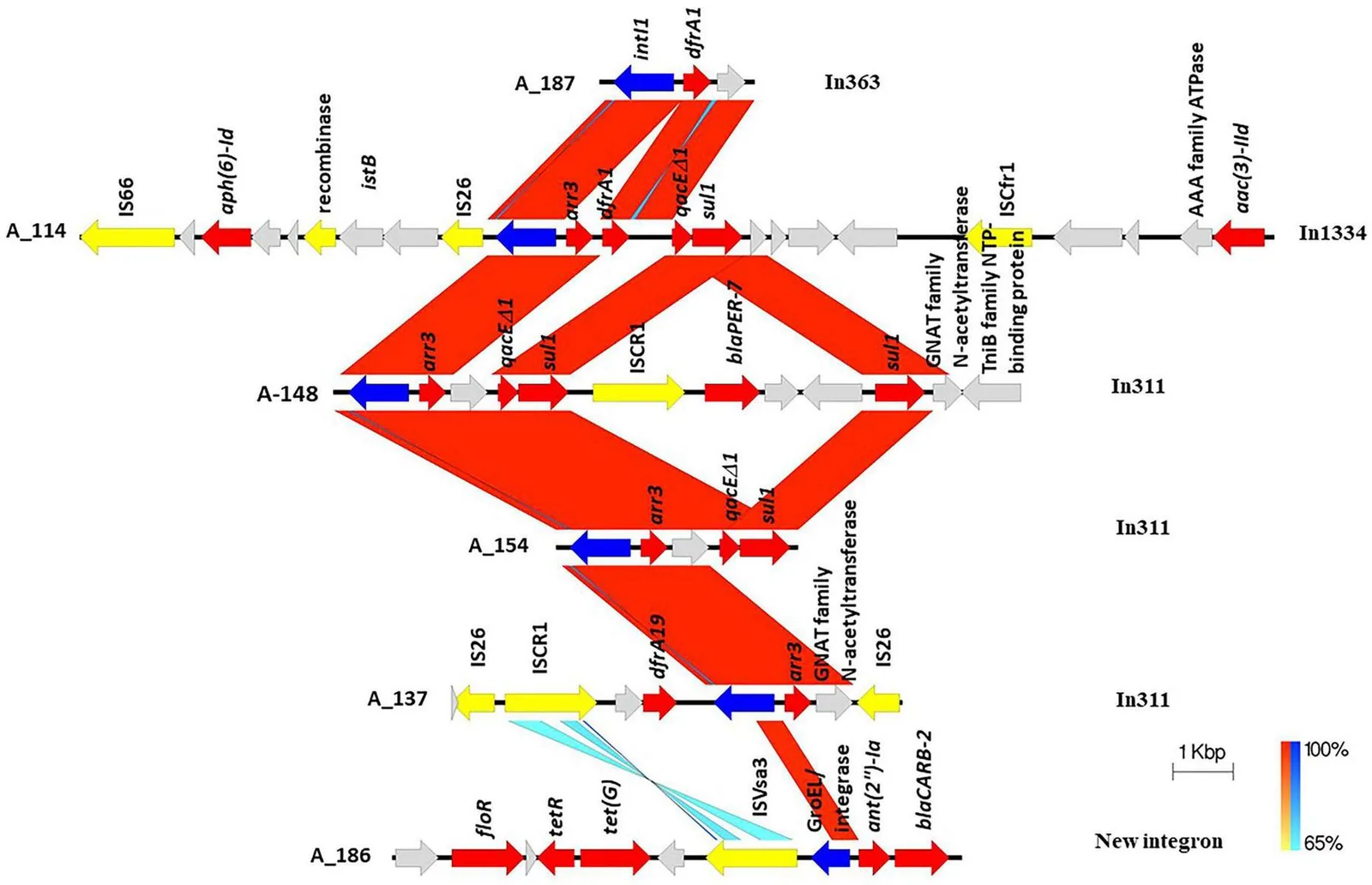
Diagrammatic representation of class 1 integron found among the *Acinetobacter* non-*baumannnii* strains (A_114, A_137, A_148, A_154, A_186, A_187). These carried resistance genes including *arr3* (ADP ribosyl transferase), *aacA4’* (aminoglycoside 6′-*N*- acetyltransferase), *dfrA1* (dihydrofolate reductases type-A)*, sul1* (sulfonamide resistant dihydropteroate synthase) etc. Some integrons were embedded within transposon-like elements or associated with insertion sequences (IS*26*).

Among the 11 Anbs, class 1 integrons (In311, In1334, In363 & a new integron) were detected in six isolates (Figure 2). Three (A_114, A_148, A_154) possessed complete integrons (with both 5′-CS and 3′-CS), while three (A_137, A_186, A_187) carried incomplete structures. These carried resistance genes including *arr3*, *aacA4’*, *dfrA1, sul1*, etc. Some integrons were embedded within transposon-like elements or associated with insertion sequences (IS*26*), indicating strong potential for horizontal gene transfer. Notably, *A. indicus* A_186 carried a GroEL/integrase fusion resembling the *Salmonella* Genomic Island 1 (Rodríguez et al., 2004), suggesting possible interspecies gene flow.

#### 3.3.4 Core genome phylogenomic analysis

**FIGURE 3:**
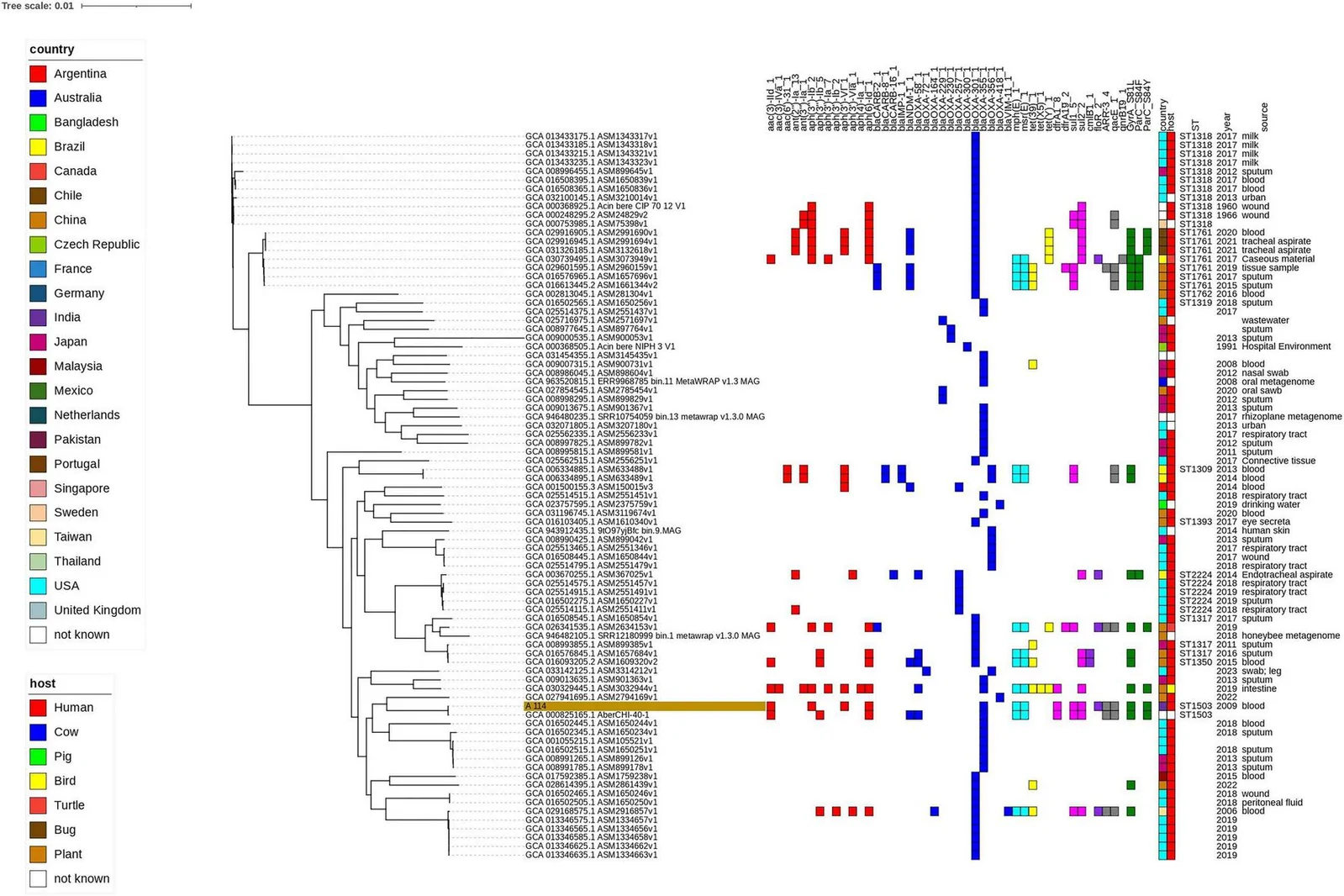
The core genome phylogenetic tree of *A. bereziniae*. The phylogenomic analysis is based on the core genome sequences of one study isolates of *A. bereziniae* (A_114) from the blood of neonate with septicemic and 83 genomes of *A. berenziniae*, downloaded from NCBI GenBank. The maximum likelihood tree was generated by IQ-TREE. Bootstrap support values were calculated from 1,000 replicates. The circles on the tree indicate bootstrap values greater than 95%. Resistance genes are colored by antibiotic class, with aminoglycoside resistance in red, β-lactam resistance in blue, macrolide resistance in sea green, tetracycline resistance in yellow, sulfamethoxazole-trimethoprim resistance in pink, phenicol resistance in purple, quaternary ammonium compounds resistance in gray and QRDR mutations in green. The presence/absence of these genes are indicated using a heat map. The names of the countries and host are on the left, and are colored in the “locate” column accordingly. The source of isolation, sample collection year and Sequence Type (ST) have also been externally incorporated into the tree phylogeny. The study isolates are denoted with a yellow color.

The core genome phylogenetic tree was constructed using 11 study Anb isolates along with publicly available genomes from NCBI GenBank: *A. schindleri* (*n* = 38), *A. indicus* (*n* = 129), *A. variabilis* (*n* = 45) and *A. bereziniae* (*n* = 83). The analysis showed that the closest strain to which the *A. bereziniae* A_114 was related was AberCHI-40-1 (GCA_000825165.1), with a SNP distance of 107. AberCHI-40-1 (isolated from pus sample, Tamil Nadu, India in 2005), carried *bla*_NDM–1_ on plasmid pNDM-40-1 (Jones et al., 2014), whereas A_114 lacked both *bla*_NDM–1_ and pNDM-40-1-like sequence. Both strains shared common resistance genes including *aac(3)IId, aph(6)Id, mph(E), msr(E), dfrA1, sulI, sulII, arr3*, *qacE* and identical QRDR mutations in GyrA (Ser81Leu) and ParC (Ser84Tyr) (Figure 3; Roy et al., 2021).

**FIGURE 4:**
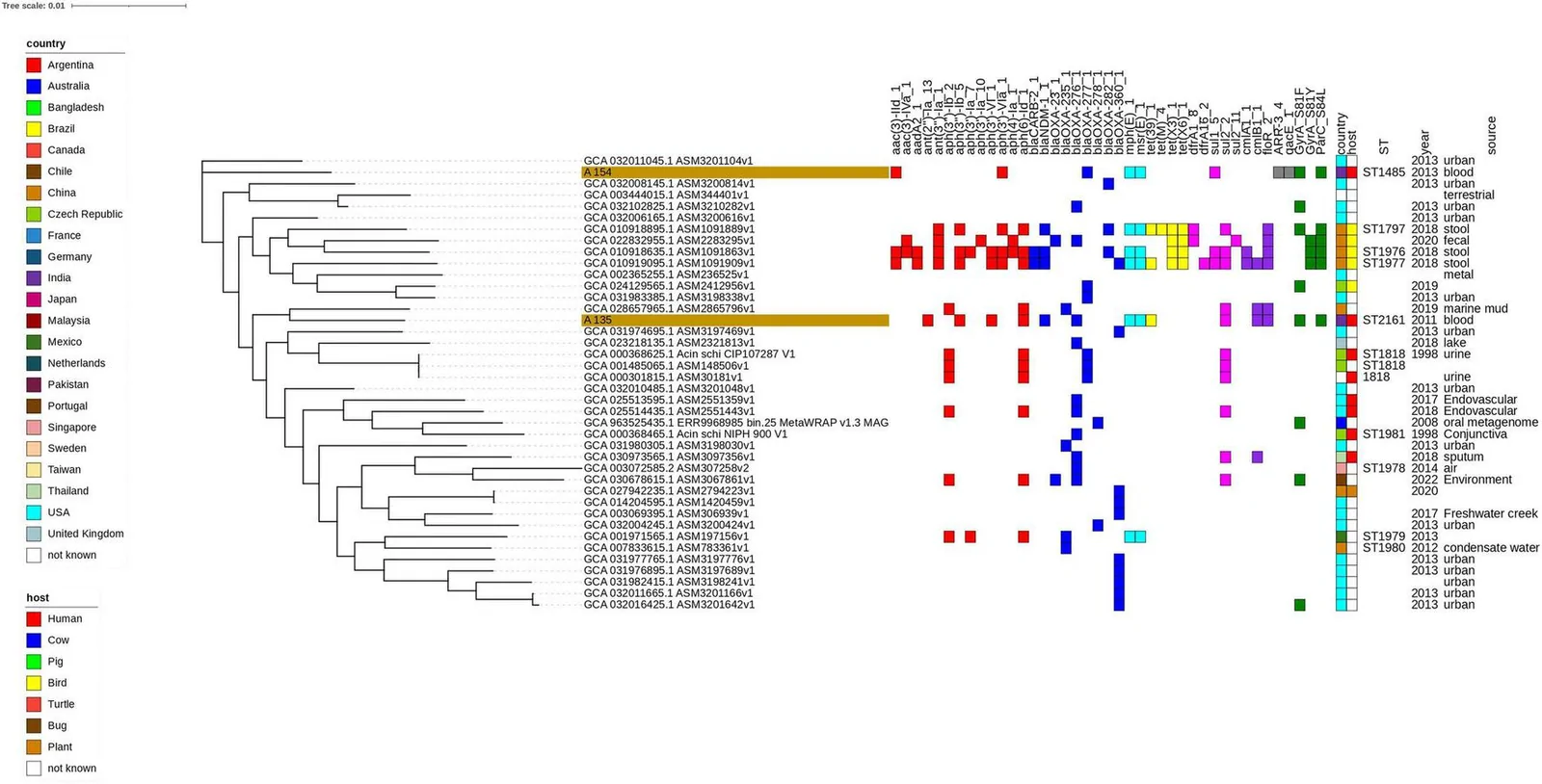
The core genome phylogenetic tree of *A. schindleri*. The phylogenomic analysis is based on the core genome sequences of two study isolates of *A. schindleri* (A_135, A_154) from the blood of neonate with septicemic and 38 genomes of *A. schindleri*, downloaded from NCBI GenBank. The maximum likelihood tree was generated by IQ-TREE. Bootstrap support values were calculated from 1,000 replicates. The circles on the tree indicate bootstrap values greater than 95%. Resistance genes are colored by antibiotic class, with aminoglycoside resistance in red, β-lactam resistance in blue, macrolide resistance in sea green, tetracycline resistance in yellow, sulfamethoxazole-trimethoprim resistance in pink, phenicol resistance in purple, quaternary ammonium compounds resistance in gray and QRDR mutations in green. The presence/absence of these genes are indicated using a heat map. The names of the countries and host are on the left, and are colored in the “locate” column accordingly. The source of isolation, sample collection year and Sequence Type (ST) have also been externally incorporated into the tree phylogeny. The study isolates are denoted with a yellow color.

*A. schindleri* A_135 and A_154 were phylogenetically distant and shared only *mph(E)* and *msr(E)* as common resistance genes. Notably, A_135 carried *bla*_NDM–1_. Among 38 *A. schindleri* genomes in GenBank, only three isolates (from Chinese goose) carried *bla*_NDM–1_. Although, human *A. schindleri* with *bla*_NDM–1_ have been reported (Montaña et al., 2018), A_135 represents the first human *A. schindleri* with *bla*_NDM–1_ subjected to whole-genome analysis (Figure 4). No close phylogenetic relationships were detected among the Anbs.

**FIGURE 5:**
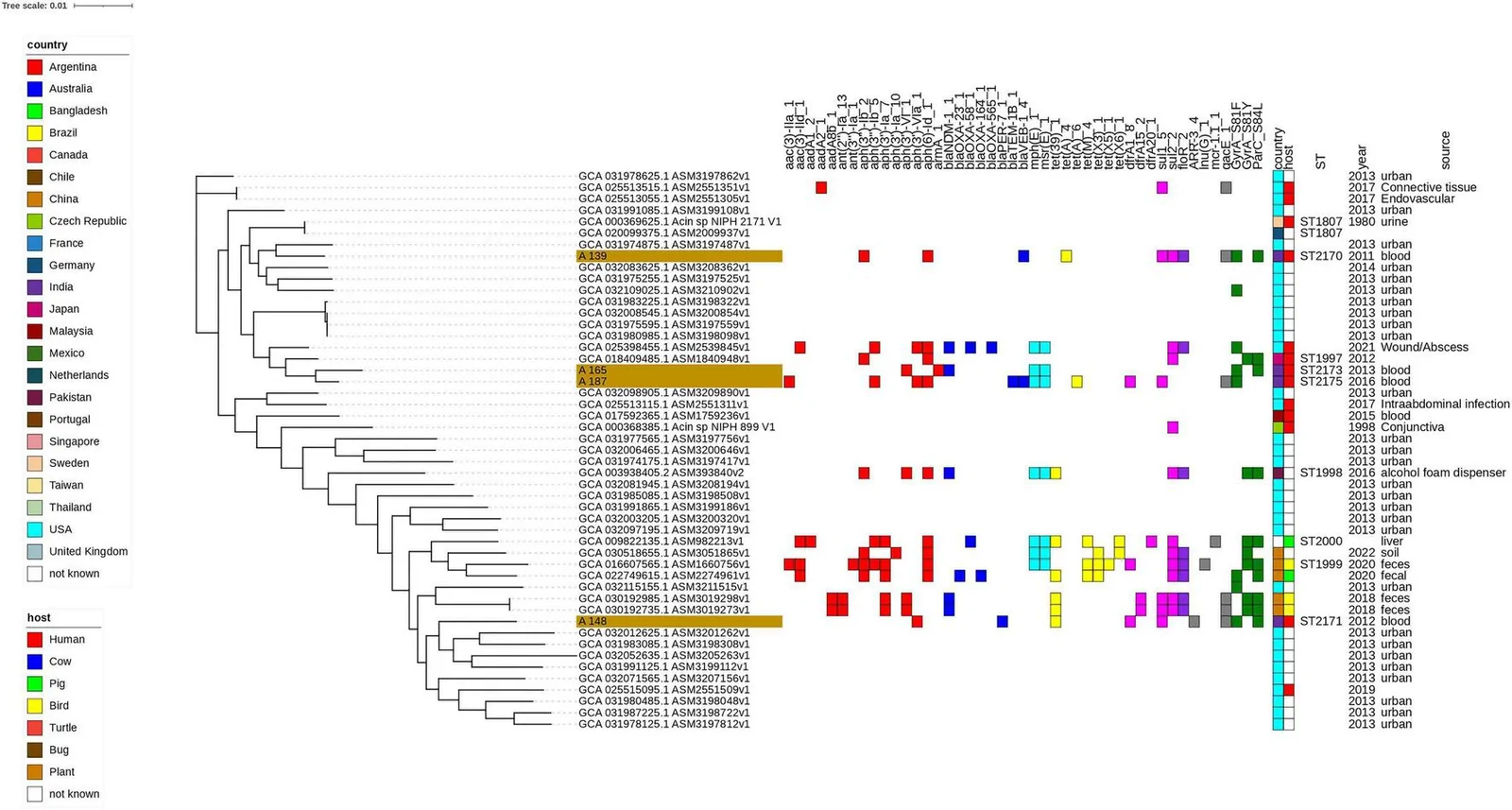
The core genome phylogenetic tree of *A. variabilis*. The phylogenomic analysis is based on the core genome sequences of four study isolates of *A. variabilis* (A_139, A_148, A_165, A_187) from the blood of neonate with septicemic and 45 genomes of *A. variabilis*, downloaded from NCBI GenBank. The maximum likelihood tree was generated by IQ-TREE. Bootstrap support values were calculated from 1,000 replicates. The circles on the tree indicate bootstrap values greater than 95%. Resistance genes are colored by antibiotic class, with aminoglycoside resistance in red, β-lactam resistance in blue, macrolide resistance in sea green, tetracycline resistance in yellow, sulfamethoxazole-trimethoprim resistance in pink, phenicol resistance in purple, quaternary ammonium compounds resistance in gray and QRDR mutations in green. The presence/absence of these genes are indicated using a heat map. The names of the countries and host are on the left, and are colored in the “locate” column accordingly. The source of isolation, sample collection year and Sequence Type (ST) have also been externally incorporated into the tree phylogeny. The study isolates are denoted with a yellow color.

Among *A. variabilis* isolates, A_165 and A_187 were closely related but distant from A_139 and A_148. Resistance profiles varied among the study isolates, with *bla*_NDM–1_ detected only in A_165 (Figure 5). No study isolates were closely related to global *A. variabilis* genomes.

**FIGURE 6:**
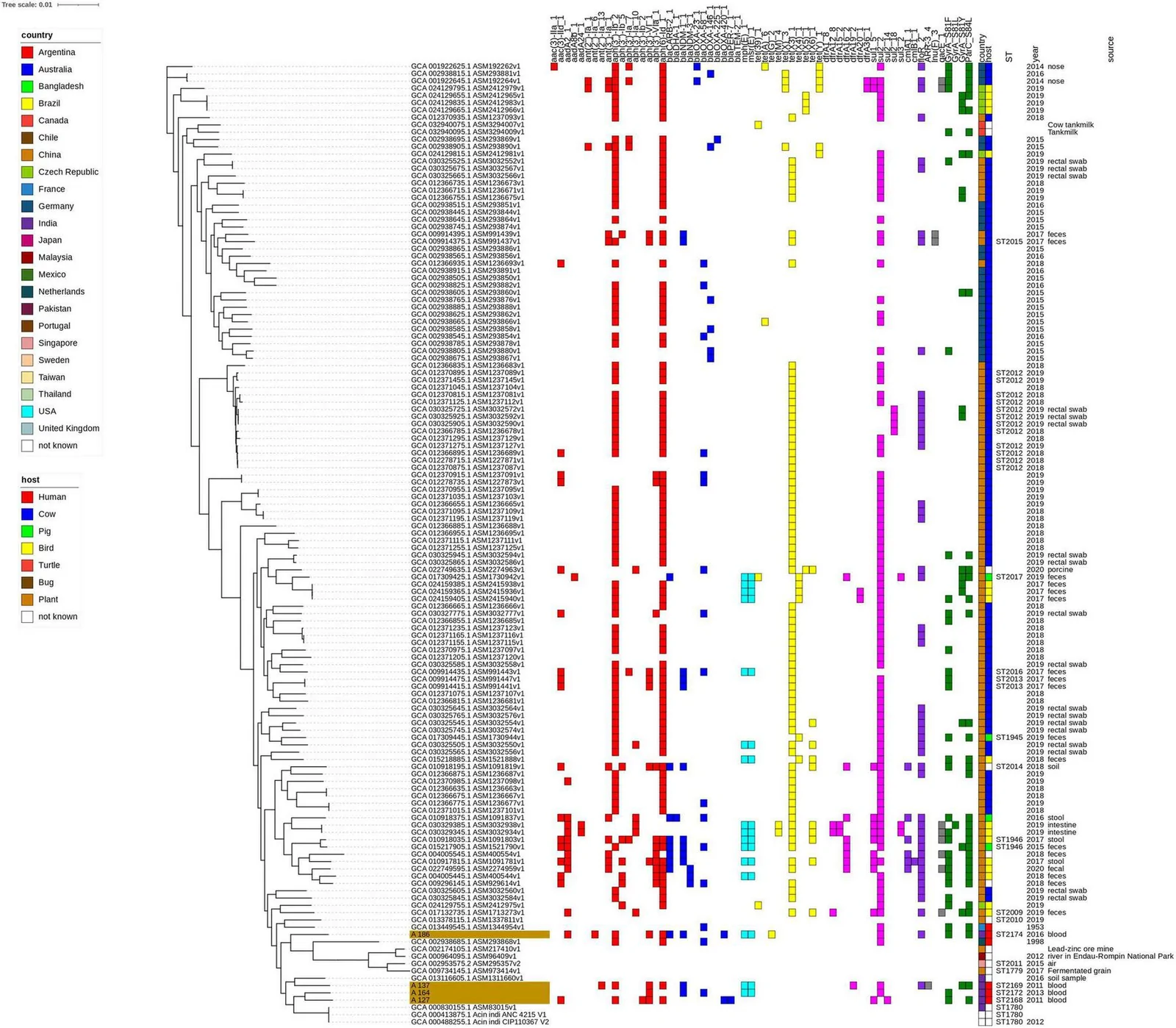
The core genome phylogenetic tree of *A. indicus*. The phylogenomic analysis is based on the core genome sequences of four study isolates of *A. indicus* (A_127, A_137, A_164, A_186) from the blood of neonate with septicemic and 129 genomes of *A. indicus*, downloaded from NCBI GenBank. The maximum likelihood tree was generated by IQ-TREE. Bootstrap support values were calculated from 1,000 replicates. The circles on the tree indicate bootstrap values greater than 95%. Resistance genes are colored by antibiotic class, with aminoglycoside resistance in red, β-lactam resistance in blue, macrolide resistance in sea green, tetracycline resistance in yellow, sulfamethoxazole-trimethoprim resistance in pink, phenicol resistance in purple, quaternary ammonium compounds resistance in gray and QRDR mutations in green. The presence/absence of these genes are indicated using a heat map. The names of the countries and host are on the left, and are colored in the “locate” column accordingly. The source of isolation, sample collection year and Sequence Type (ST) have also been externally incorporated into the tree phylogeny. The study isolates are denoted with a yellow color.

*A. indicus* strains A_127, A_137, A_164 and A_186 were closely related but showed distinct resistance profiles. Among 129 *A. indicus* genomes in GenBank, most were bovine-derived, while only two human-derived strains (GCA_013449545.1, GCA_002938685.1) clustered closely with the study isolates (32,420–55,145 SNPs). Bovine strains, primarily from China and Germany, formed a separate clade, indicating phylogenetic divergence between human- and bovine-associated *A. indicus*. The Indian environmental strain *A. indicus* CIP 110367 (GCA_000830155.1) (Malhotra et al., 2012) was also closely related to the study strains (30212-31951 SNPs). Interestingly, this strain was phylogenetically closer to the human isolates (30212-36859 SNPs) than to bovine strains, suggesting possible adaptation or shared ancestry (Figure 6).

Overall, core genome phylogenetic analysis indicated that the study *Acinetobacter* non-*baumannii* isolates were genetically diverse and did not form a single clonal cluster. While a few isolates showed similarity to strains in public databases, most formed distinct lineages. ARGs profiles were heterogeneous, with carbapenemases such as *bla*_NDM–1_ and *bla*_OXA–58–*like*_ detected sporadically, while other genes (aminoglycoside, tetracycline, sulfonamide and macrolide resistance) were widely distributed. Conserved QRDR mutations in *gyrA* and *parC* were detected, indicating selective pressures across different ecological niches. Core-genome analysis revealed that resistance gene content varied considerably even among the closely related strains, indicating horizontal gene transfer rather than clonal expansion.

### 3.4 *In vitro* pathogenicity of the novel Anb isolates

#### 3.4.1 Biofilm formation

**FIGURE 7:**
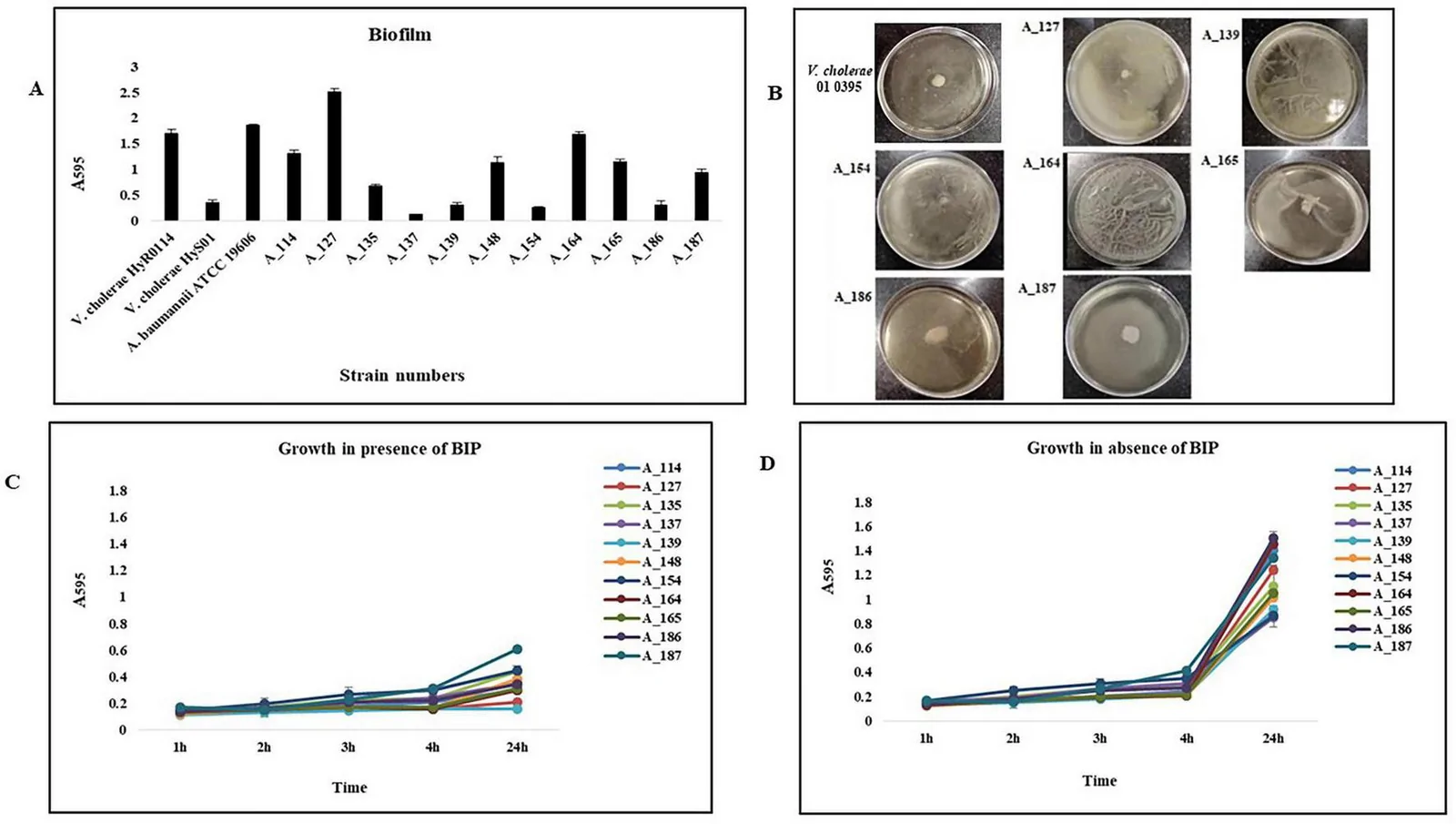
Virulence potential of 11 *Acinetobacter* non-*baumannnii* strains belonging to novel sequence types. **(A)** *In vitro* study of biofilm production. *Vibrio cholerae* HyRS01 was used as low biofilm producer and *Vibrio cholerae* HyR0114 and *A. baumannii* ATCC 19606 were used as high biofilm producer. Bars represent the average of two separate assays, with error bars representing the standard deviation. **(B)** Surface motility assay on low percentage (0.3%) LB agar plates. *Vibrio cholerae* 01 0395 was used as positive control (upper left) and seven studied strains showed positive results for motility. **(C)** Growth at 37°C in presence of the iron chelator 2,2’-bipyridyl (Bip) at a final concentration of 150 mM. **(D)** Growth at 37°C in absence of Bip. Growth was quantified by measuring the OD_595_ nm at 1, 2, 3, 4, and 24 h. All assays were performed in duplicate.

All 11 Anb isolates harbored *ompA* and the *bfmRS* two-component regulatory system, both of which are essential for biofilm formation and pilus expression (Roy et al., 2022; Upmanyu et al., 2022). However, the universal presence of these genes did not correspond to uniformly high biofilm production among the isolates. Six out of the eleven Anb strains exhibited strong biofilm formation, with OD_595_ values ranging from (0.95 ± 0.05) to (2.509 ± 0.07) (Supplementary Table 1) compared with the negative control *V. cholerae* HyRS01 (OD_595_ = 0.37 ± 0.04). The level of biofilm production in these isolates was comparable to that of the positive controls *V. cholerae* HyR0114 (OD_595_ = 1.7 ± 0.08) and *A. baumannii* ATCC19606 (OD_595_ = 1.9 ± 0.01) (Table 3, Figure 7A, Supplementary Table 1). Additional biofilm-associated determinants, including c*suABCDE*, *pgaABCD*, and *bap*, were variably present among the isolates; however, their presence did not consistently correlate with the observed biofilm phenotypes. For instance, the *csuCDE* operon, which encodes components of the chaperone-usher secretion system involved in surface attachment and microcolony formation (Roy et al., 2022; Upmanyu et al., 2022), was detected in *A. bereziniae* A_114 and *A. indicus* A_164, both of which were strong biofilm producers. In contrast, the *pgaABCD* operon, synthesizing extracellular poly-β-1,6-N-acetylglucosamine, a key determinant of biofilm formation (Roy et al., 2022; Upmanyu et al., 2022), was identified in six isolates among which only *A. indicus* A_127 and A_164 produced robust biofilms. Likewise, the biofilm-associated protein gene (*bap*), which contributes to mature biofilm architecture and adhesion to both biotic and abiotic surfaces (Roy et al., 2022; Upmanyu et al., 2022), was detected in *A. schindleri* A_135 and A_154; however, these isolates exhibited only moderate and weak biofilm formation, respectively. These observations highlight substantial genotype-phenotype variability in biofilm formation among the studied isolates. This discrepancy may be influenced by factors such as differential gene regulation, post-transcriptional control mechanisms, strain-specific genomic backgrounds, or environmental conditions affecting gene expression.

**TABLE 3:** Assessment of *in vitro* and *in vivo* virulence and the associated virulence factors in the novel *Acinetobacter* non-*baumannii* strains (*n* = 11).

Strain number	Organism	Biofilm forming capability	Biofilm genes *ompA* *bfmRS* *csuABCDE* *pgaABCD* *bap*	Motility capability	Motility genes *pilU* *pilT*	Growth under iron-limiting condition after 24 h (OD_595_)	Iron acquisition genes *bauABCDE*	Adherence capability	Invasion capability	Adherence/ invasion genes *ompA* *csuABCDE* *bla*_PER_ *bap* *pilU* *pilT*	Apoptosis (Percentage of dead cells)	% of survival in *in vivo* mice model
A_114	*A. bereziniae*	Strong	+++ −−	No	−−	Reduced growth	+	Yes	Yes	++−−−−	27.05	66.66
A_127	*A. indicus*	Strong	++−+−	Yes	−+	Reduced growth	−	Yes	No	+−+−− +	32.25	33
A_135	*A. schindleri*	Moderate	++−++	No	+−	Reduced growth	−	Yes	Yes	+−−++−	14.7	100
A_137	*A. indicus*	Low	++−+−	No	−+	Reduced growth	−	Yes	No	+−−−−+	6.55	66.66
A_139	*A. variabilis*	Low	++−−−	Yes	+−	Reduced growth	−	Yes	No	+−−−+−	18.6	83
A_148	*A. variabilis*	Strong	++−−−	No	+−	Reduced growth	−	Yes	No	+−+−+−	20.65	100
A_154	*A. schindleri*	Low	++−++	Yes	+−	Reduced growth	−	No	No	+−−++−	34.6	100
A_164	*A. indicus*	Strong	++++ −	Yes	−+	Reduced growth	−	No	No	++−−−+	31.4	83
A_165	*A. variabilis*	Strong	++−−−	Yes	+−	Reduced growth	−	Yes	Yes	+−−−+−	20.05	100
A_186	*A. indicus*	Low	++−+−	Yes	−+	Reduced growth	−	No	No	+−−−−+	19.55	83
A_187	*A. variabilis*	Strong	++−−−	Yes	+−	Reduced growth	−	No	No	+−−−+−	22.65	50

“+” Anb strains possessed the respective virulence genes. “−” Anb strains did not possess the respective virulence genes. Strong biofilm formation was considered when the readings were at least three times greater than the negative control (Supplementary Table 1). Reduced growth at 24 h under iron-limiting conditions indicated OD_595_ = 0.16 ± 0.004 to 0.608 ± 0.007 (Supplementary Table 1).

#### 3.4.2 Motility

Most of the 11 Anb isolates exhibited surface motility within 24–72 h, except *A. bereziniae* A_114, *A. schindleri* A_135, *A. indicus* A_137 and *A. variabilis* A_148 (Figure 7B). Although *Acinetobacter* spp. lack flagella, they display surface-associated or twitching motility mediated by type IV pili (Corral et al., 2021). Key regulatory genes (*pilR, pilS, pilT, pilU*) were variably present among the Anb isolates including *pilU* which was detected in four *A. variabilis* and two *A. schindleri* and *pilT* in four *A. indicus* (Table 3). Despite possessing these genes, strains such as *A. schindleri* A_135, *A. indicus* A_137 and *A. variabilis* A_148 were non-motile, suggesting regulatory or expression-level differences rather than presence or absence of the genes.

#### 3.4.3 Growth under iron-limiting condition

All 11 Anb isolates showed reduced growth at 24 h under iron-limiting conditions (OD_595_ = 0.16 ± 0.004–0.608 ± 0.007) in the presence of the iron chelator 2,2’-bipyridyl (Table 3, Figures 7C,D, Supplementary Table 1), indicating absence of siderophore system. Notably, only *A. bereziniae* A_114 carried the acinetobactin-mediated iron acquisition system *bauABCDE* which is a major siderophore locus in *A. baumannii* (Sheldon and Skaar, 2020) but still exhibited reduced growth. This again implies possible regulatory differences rather than presence or absence of the gene.

#### 3.4.4 Analysis of adherence, invasion and apoptosis

**FIGURE 8:**
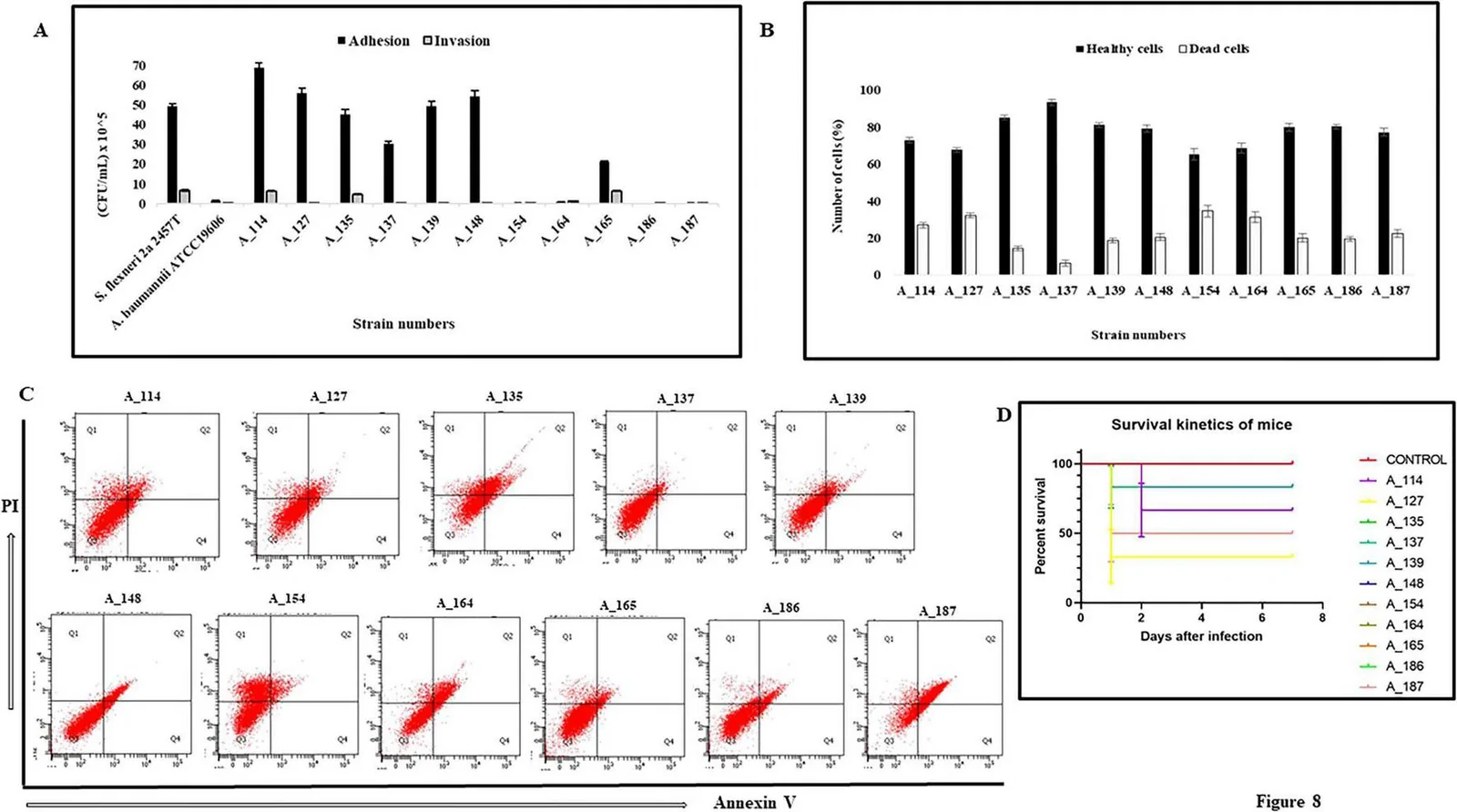
Virulence potential of 11 *Acinetobacter* non-*baumannnii* strains belonging to novel sequence types. **(A)** *In vitro* Adherence/invasion experiments. A549 cells were infected for 90 min with Anbs and the number of adherent/invasive bacteria are expressed as CFU/mL. Experiments were performed in duplicate. Error bars represent standard deviation. **(B,C)** Quantitative evaluation of % of dead cells (apoptosis and necrosis) and healthy cells was performed by using the flowcytometry methods of double staining using an FITC-conjugated annexin-V/ propidium iodide (PI). Bars represent the average of two separate assays, with error bars representing the standard deviation. **(D)** The survival kinetics of mice infected with Anb strains were analyzed and presented using Kaplan–Meier survival curves generated in GraphPad Prism v8.0.1.

Seven out of 11 Anb isolates showed good adherence on A549 epithelial cells (CFU/mL: 21.3 x 10^5^± 0.29–69 × 10^5^± 2.5) when compared with the positive control *S. flexneri* 2a2457T (CFU/mL: 49.4 × 10^5^± 1.5). However, 3 (*A. bereziniae* A_114, *A. schindleri* A_135, *A. variabilis* A_165) of these 7 Anbs demonstrated invasion (CFU/mL: 4.7 × 10^5^± 0.23 to 6.5 × 10^5^± 0.29) when compared with the positive control (CFU/mL: 6.7 × 10^5^± 0.04) (Table 3, Figure 8A, Supplementary Table 1). Several factors known to contribute to adhesion or invasion in *A. baumannii*, including OmpA, the CsuA/BABCDE pilus system, Bap, PER-1, and Type IV pili (encoded by *pil* genes) (Giltner et al., 2012; Roy et al., 2022; Upmanyu et al., 2022), were detected among the Anb isolates. However, their presence did not correlate with the ability of the strains to adhere to or invade epithelial cells. For instance, although OmpA was present in all isolates, adhesion and invasion were not observed in every case. Similarly, *pil* genes were detected in almost all isolates; however, adhesion was not observed in all cases. On the other hand, CsuABCD and Bap were detected in the isolates A_114 and A_135, respectively, both of which exhibited successful adhesion. These observations suggest that, in addition to these genes, other factors may contribute to adhesion and invasion, and that the presence of these known determinants alone is insufficient to confer these phenotypes.

To comprehensively evaluate the pathogenic potential of the studied Anb isolates, apoptosis assays were also performed. While adhesion and invasion reflect the ability of isolates to colonize and penetrate host cells, apoptosis assays provide insight into their capacity to induce host cell damage. Therefore, quantifying apoptosis in infected A549 epithelial cell lines serves as an indicator of the pathogenic potential of different isolates. Analysis of apoptosis indicated that the percentage of dead cells among the studied Anb isolates (apoptotic plus necrotic cells) ranged from 6.6 to 34.6% (Table 3, Figures 8B,C, Supplementary Table 1). Most isolates induced low to moderate cytotoxicity ( < 30% cell death) in host cells. Previous studies on *A. baumannii* have reported substantially higher levels of epithelial cell apoptosis, often reaching 18–65% within 24 h (Krzymiñska et al., 2012; Rafiei Delfan et al., 2024). This effect has been attributed largely to virulence factors such as OmpA, which can induce mitochondrial damage and activate apoptotic pathways in host cells (Zhao et al., 2026). In contrast, despite the presence of OmpA in all the studied isolates, the relatively lower levels of apoptosis observed here suggest that Anb isolates may require additional virulence determinants to induce cytotoxic effects.

### 3.5 *In vivo* virulence potential of Anb strains

Virulence potential was assessed using a 7-day mouse survival model. Seven of the 11 Anb strains (*A. schindleri* A_135 and A_154; *A. variabilis* A_139, A_148 and A_165; *A. indicus* A_164 and A_186) exhibited low virulence with 83–100% survival of the infected mice. Two strains (*A. bereziniae* A_114 and *A. indicus* A_137) showed moderate virulence (66% survival). In contrast, two other strains (*A. indicus* A_127 and *A. variabilis* A_187) demonstrated comparatively higher virulence as shown by reduction in survival (33–50%). Notably, these two strains did not harbor the highest number of VFs. In fact, their virulence gene content was comparable to those of other Anb strains that did not cause mortality in mice (Table 3). Overall, compared to *A. baumannii*, which typically exhibited high lethality in murine models under similar experimental conditions (Wang et al., 2024), most of the studied Anb strains displayed lower virulence.

Collectively, *in vitro* and *in vivo* virulence assays highlighted no consistent correlation between the virulence gene repertoire, *in vitro* pathogenicity and *in vivo* lethality as also reported in an earlier study on Anbs (Yaikhan et al., 2024). Most of the studied Anb strains which showed neonatal fatality, didn’t show adhesion and invasion capabilities, and all showed attenuated lethality in the murine infection model. Similarly, isolates which demonstrated comparatively higher virulence in mice, did not display invasion capacity and were not associated with neonatal mortality. Consistent with these observations, our earlier work on two *A. pittii* (Roy et al., 2024) isolates also demonstrated high mortality in the murine model without successful invasion and only one was associated with neonatal fatality. Together, these observations indicate a clear discordance between experimental virulence phenotypes and clinical outcomes, reinforcing the fact that Anb infection is multifactorial and not solely determined by the presence and absence of the known VFs.

## 4 Conclusion

Anb studies are becoming more important because of their ability to acquire antimicrobial resistance genes, resulting in a growing number of reports of human infections in both adults and neonates and our findings further support these observations. In addition, the mechanisms of virulence of these species remain poorly understood and require further investigation. In the present study, Anb isolates accounted for only 11% of all *Acinetobacter* infections in the neonatal unit during the study period, indicating that their prevalence remains considerably lower than that of *A. baumannii*.

The identification of Anb isolates at the species level has been challenging, which likely contributed to the under-recognition of infections caused by these organisms. However, the introduction of MALDI-TOF MS, *rpoB* gene sequencing, and WGS has substantially improved the accurate identification of Anb species. Despite these technological advances, genomic characterization of Anb isolates remains limited compared with *A. baumannii*, highlighting the need for continued genome-based analyses to identify emerging lineages and better understand their epidemiology.

In line with the earlier studies, our results also indicated that the most common carbapenemase genes detected in the Anb species are *bla*_NDM–1_ and *bla*_OXA–58–like_ genes either alone or in combination. Considering that *bla*_NDM–1_ is endemic in India, its detection in clinical isolates is not unexpected. Furthermore, the conserved genetic contexts surrounding *bla*_NDM–1_ across different *Acinetobacter* species suggest that Anb species are progressively acquiring this gene from *A. baumannii* through horizontal gene transfer, thereby increasing their potential to cause healthcare-associated infections. Notably, the carbapenemase genes identified in the present study were most likely integrated into the chromosome, as has also been reported previously in *Acinetobacter* species. Chromosomal integration may facilitate the long-term persistence of these resistance determinants, allowing them to be maintained under antibiotic selective pressure in hospital environments and potentially disseminated to other bacterial species through mobile genetic elements.

Core genome analysis revealed considerable genomic diversity among the Anb isolates. Although the distribution of *bla*_NDM–1_ and *bla*_OXA–58–like_ genes was relatively limited, several other antimicrobial resistance determinants, particularly genes encoding aminoglycoside-modifying enzyme were widely distributed across different species.

In *in vitro* and *in vivo* virulence assessments, no consistent correlation between virulence gene repertoire, *in vitro* pathogenicity and *in vivo* lethality was detected among the studied Anb genomes. These results reinforce the concept that Anb pathogenicity is probably multifactorial and that additional virulence determinants, not identified in the present study, may contribute to their pathogenic potential which warrants more in-depth investigations. It should also be noted that, the virulence determinants and pathogenicity mechanisms of Anb species are still poorly characterized, and the reliability of current databases (e.g., VFDB) for these organisms remains uncertain. Therefore, the lack of a clear association between virulence gene repertoire and the observed phenotypic traits in the present study may also reflect current limitations in the understanding and annotation of Anb virulence factors rather than a true absence of underlying biological relationships.

Despite the variability in virulence traits, the presence of multiple antimicrobial resistance determinants, together with their colonization abilities, suggests a significant potential for persistence and dissemination of these organisms in hospital environments under antibiotic selective pressure. Overall, this study highlights the emerging clinical significance of multidrug-resistant Anb species. Continuous genomic surveillance, accurate species identification, and strengthened antimicrobial stewardship will be essential to monitor and control the spread of these under-recognized pathogens in healthcare settings.

## Data Availability

The genomes of 11 *Acinetobacter* non-*baumannii* isolates were deposited in NCBI under the BioProject number PRJNA762653, with genome accession numbers JAKENB000000000, JAJADB000000000, JAJADD000000000, JAJADE000000000, JAJADF000000000, JAKENC000000000, JAJADH000000000, JAJADM000000000, JAJADN000000000, JAJADP000000000, and JAKENE000000000.

## Footnotes

1
https://www.bacterio.net/genus/acinetobacter
