# Supplementary material for: Deciphering the genomic landscape of novel Acinetobacter non-baumannii lineages causing neonatal septicemia: carbapenem resistance and virulence
Source: Front Microbiol. 2026 Aug 24;17:1889965. doi: 10.3389/fmicb.2026.1889965 (PMC13548351; doi:10.3389/fmicb.2026.1889965)
Supplement: Supplementary file 1 [file Data_Sheet_1.zip › Datasheet 1/Supplementary Methods.docx]

**Deciphering the Genomic Landscape of Novel** ***Acinetobacter* non-baumannii Lineages Causing Neonatal Septicemia: Carbapenem Resistance and Virulence**

Subhasree Roy,^a^ Daichi Morita,^b^ [Tanusree](https://pubmed.ncbi.nlm.nih.gov/?term=%22Veeraraghavan%20B%22%5BAuthor%5D) Das,^a^ Sanjib Das,^a^ Souvik Nandy,^a^ Bijan Saha,^c^ Sulagna Basu ^a*^

^a^Division of Bacteriology, ICMR-National Institute for Research in Bacterial Infections (ICMR-NIRBI), Kolkata, India

^b^Department of Microbiology, Graduate School of Biomedical and Health Sciences, Hiroshima University, Hiroshima, Japan

^c^Department of Neonatology, Institute of Post-Graduate Medical Education & Research and SSKM Hospital, Kolkata, India

**Materials and methods**

***In Vitro* Virulence Assays**

**Biofilm formation assay**

Biofilm formation of the Anb isolates was assessed as previously described. Overnight LB broth cultures were used to inoculate fresh LB broths which were incubated at 37 °C with shaking until the OD600 reached to 0.5 MacFarland. 200 µL of this bacterial suspension were placed into wells of a Ushaped polystyrene 96-well plate. After incubation at 37^0^C for 24 h, the wells were washed twice by immersion in distilled water, followed by the addition of 200 µL of a 0.5% crystal violet solution. It was kept for 20 min at room temperature, washed three times with distilled water. After drying at 37°C for 1 h, bound crystal violet was dissolved with 200 µL of 95% ethanol (v/v). Biofilm quantification was determined by measuring the absorbance at 595 nm (A_595_). Strong biofilm was considered when the readings were at least three times greater than the negative control. All assays were performed in duplicate. *Vibrio cholerae* HyRS01 and *Vibrio cholerae* HyR0114 were used as low biofilm producer and high biofilm producer respectively (**Mukherjee *et al*., 2020, Chowdhury *et al*., 2016**).

**Motility assays**

Surface motility was assessed on low percentage agar plates. Two microlitres of overnight LB broth cultures were placed in the center of a Luria Bertani plate containing 0.3% agarose. Plates were allowed to dry for 30 minutes in a laminar flow hood and then incubated in at 37°C with 80% of humidity. The radii of surface extension were measured at 24, 48 and 72 hours of incubation. All assays were performed in duplicate. *Vibrio cholerae* 01 0395 was used as positive control in motility assay. Experiments were run in duplicate (**Chowdhury *et al*., 2016, Carretero-Ledesma *et al*., 2018**).

**Growth in iron limiting conditions**

Freshly prepared cultures of each strain were adjusted to a concentration of 0.5 MacFarland CFU/mL in Mueller-Hinton broth with or without the iron chelator 2,2’-bipyridyl (Bip) at a final concentration of 150 mM. Two-hundred µL of the bacterial suspensions were added to wells of 96-well flat bottom polystyrene microplates and growth at 37°C was quantified by measuring the OD_595nm_ at 1, 2, 3, 4 and 24 hours. All assays were performed in duplicate (**Carretero-Ledesma *et al*., 2018**).

**Cell Adherence/Invasion Assays**

The adherence/invasion assay was performed as earlier. Briefly, 3.83 X 10^5^ A549 cells (human lung tumor cells) were deposited onto 12-well polystyrene plates. Twenty-four hours before infection, the culture media was changed by DMEM (Dulbecco′s Modified Eagle′s Medium) without antibiotics and serum. The cells were incubated with *Acinetobacter* strains at a MOI of 100. The infected plates were centrifuged for 4 min at 200 X g prior to the incubation to promote adherence of bacteria to cells and to synchronize infections. Infected monolayers were then incubated at 37^°^C with 5% CO2 for 90 min. To study the invasion, after infection A549 monolayer was incubated with DMEM containing gentamicin (300 µg/ml) for 2 h at 37^o^C to kill extracellular bacteria. The cells were then washed three times with PBS (pH 7.4). Cells were immediately lysed with PBS-Triton X-100 1% to release adhered and intracellular Anb from A549 cells. To quantify intracellular bacteria (CFUs), serial dilution 1:10 or 1:100 were made and plated onto LB agar plates was performed. These set of experiments were performed in duplicate on 2 different days. *S. flexneri* 2a2457T and *A. baumannii* ATCC 19606 were used as positive and negative controls, respectively. The number of invasive bacteria was expressed as the total number of colony forming units (CFU) associated bacteria per well after gentamicin treatment (**Lázaro-Díez *et al*., 2016**).

**Detection of apoptosis**

Quantitative evaluation of apoptosis was performed by using the flowcytometry methods of double staining using an FITC-conjugated annexin-V/propidium iodide (PI) staining according to the manufacturer’s protocol (BD diagnostics). Adherent A549 cells were infected with Anb strains at a MOI of 100 for 24 h, and then detached, washed with PBS and incubated with PI and Annexin-V for 15 min at 37 °C. Excess PI and Annexin-V were then washed with PBS and cells were analyzed by FACS Aria II using BD FACSCanto^TM^ Clinical Software v.3 (Becton Dickinson).

**References**

Carretero-Ledesma M, García-Quintanilla M, Martín-Peña R et al. Phenotypic changes associated with Colistin resistance due to Lipopolysaccharide loss in *Acinetobacter baumannii*. Virulence 2018; 9: 930-42.  doi: 10.1080/21505594.2018.1460187.

Chowdhury G, Bhadra RK, Bag S et al. Rugose atypical Vibrio cholerae O1 El Tor responsible for 2009 cholera outbreak in India. *J Med Microbiol* 2016; 65: 1130-36. doi: 10.1099/jmm.0.000344.

Lázaro-Díez M, Navascués-Lejarza T, Remuzgo-Martínez S et al. *Acinetobacter baumannii* and *A. pittii* clinical isolates lack adherence and cytotoxicity to lung epithelial cells in vitro. Microbes Infect. 2016 Sep;18(9):559-64. doi: 10.1371/journal.pone.0037024.

Mukherjee S, Naha S, Bhadury P et al. Emergence of OXA-232-producing hypervirulent *Klebsiella pneumoniae* ST23 causing neonatal sepsis. *J Antimicrob Chemother* 2020; 75: 2004-06. doi: 10.1093/jac/dkaa080.
